# Promoting advance care planning among community-based older adults: a pilot project in Chinese society

**DOI:** 10.1093/geroni/igaf122.2618

**Published:** 2025-12-31

**Authors:** Shiyu Lu, Ning Fan, Yan Zhang

**Affiliations:** The University of Hong Kong, Hong Kong, China (People’s Republic); Forget Thee Not, Hong Kong, China (People’s Republic); The University of Hong Kong, Hong Kong, China (People’s Republic)

## Abstract

While promoting advanced care planning is a priority, knowledge remains unclear about how to prepare the aging community for advanced care planning (ACP) at an early stage (EoL), particularly in Chinese societies where discussions about end-of-life care arrangements are often considered taboo. This study evaluates the effectiveness of the Community Care Plan (CCP) pilot project in Hong Kong, which is a compassionate community service model for EoL care focusing on enhancing quality of life (“good life”) and establishing ACP for older individuals facing health deterioration (“good death” arrangements). The CCP pilot project is built on multidisciplinary collaborative services, including a professional team specializing in ACP, home-visit services by nursing teams, social workers from traditional community centers for older adults, and active community volunteers. The evaluation study employs a single-group pre- and post-test design to assess effectiveness. Following an 8-month pilot project, the mixed linear regression found that 63 eligible older adults showed improvements: an increased sense of community (β = 0.53, p = 0.008), enhanced ACP knowledge (β = 0.42, p < 0.001), more conversations with others (β = 0.16, p = 0.001), and increased readiness and self-efficacy in ACP engagement (e.g., discussing medical treatment at end-of-life with doctors and signing advanced directives). The 70 involved active community volunteers also reported improvements in quality of life, sense of community, ACP knowledge, and increased comfort initiating EoL conversations with others. The study discusses potential policy and practice implications for ACP promotion based on the preliminary findings.

